# Supplementary figures and images for: A concerted probiotic activity to inhibit periodontitis-associated bacteria
Source: PLoS One. 2021 Mar 5;16(3):e0248308. doi: 10.1371/journal.pone.0248308 (PMC7935250; doi:10.1371/journal.pone.0248308)

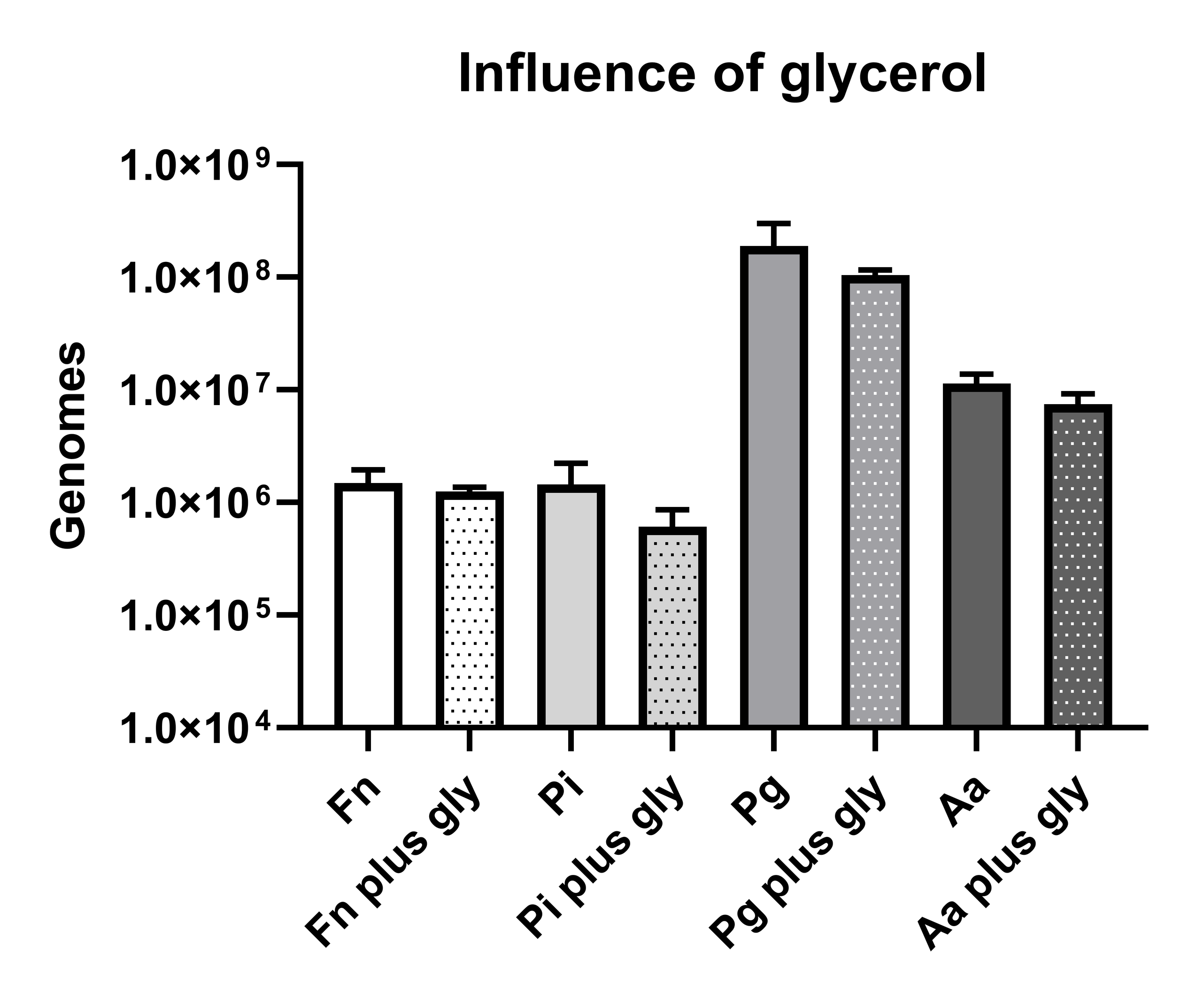

Supplement: S1 Fig — Abbreviations: Pi (Prevotella intermedia), Pg (Porphyromonas gingivalis), Fn (Fusobacterium nucleatum), Aa (Aggregatibacter actinomycetemcomitans), gly (glycerol). (TIF) [file pone.0248308.s001.tif]

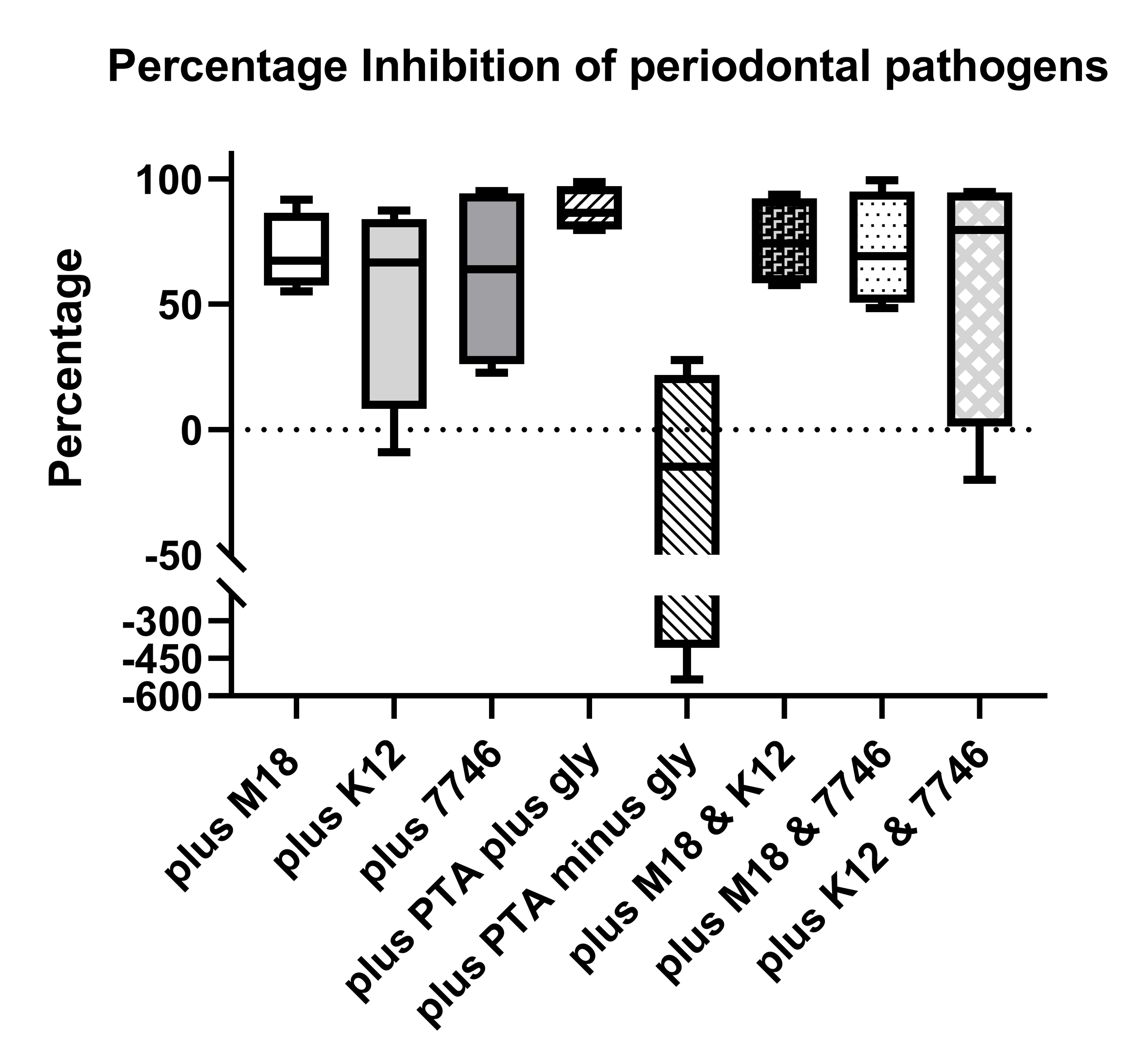

Supplement: S2 Fig — In a culture without glycerol, L. reuteri caused a growth spurt (negative inhibition) of some pathogens. For data see Table 2. Abbreviations: M18 (S. salivarius subsp. salivarius M18), K12 (S. salivarius subsp. salivarius K12), PTA (Lactobacillus reuteri ATCC PTA 5289), 7746 (Streptococcus oralis subsp. dentisani 7746), gly (glycerol). (TIF) [file pone.0248308.s002.tif]
